# Communications Between the Endoplasmic Reticulum and Other Organelles During Abiotic Stress Response in Plants

**DOI:** 10.3389/fpls.2019.00749

**Published:** 2019-06-12

**Authors:** Linchuan Liu, Jianming Li

**Affiliations:** ^1^State Key Laboratory for Conservation and Utilization of Subtropical Agro-Bioresources, South China Agricultural University, Guangzhou, China; ^2^Guangdong Key Laboratory for Innovative Development and Utilization of Forest Plant Germplasm, College of Forestry and Landscape Architecture, South China Agricultural University, Guangzhou, China; ^3^Department of Molecular, Cellular, and Developmental Biology, University of Michigan, Ann Arbor, MI, United States

**Keywords:** membrane contact sites, endoplasmic reticulum, unfolded protein response, lipid exchange and transport, calcium homeostasis, reactive oxygen species

## Abstract

To adapt to constantly changing environmental conditions, plants have evolved sophisticated tolerance mechanisms to integrate various stress signals and to coordinate plant growth and development. It is well known that inter-organellar communications play important roles in maintaining cellular homeostasis in response to environmental stresses. The endoplasmic reticulum (ER), extending throughout the cytoplasm of eukaryotic cells, is a central organelle involved in lipid metabolism, Ca^2+^ homeostasis, and synthesis and folding of secretory and transmembrane proteins crucial to perceive and transduce environmental signals. The ER communicates with the nucleus *via* the highly conserved unfolded protein response pathway to mitigate ER stress. Importantly, recent studies have revealed that the dynamic ER network physically interacts with other intracellular organelles and endomembrane compartments, such as the Golgi complex, mitochondria, chloroplast, peroxisome, vacuole, and the plasma membrane, through multiple membrane contact sites between closely apposed organelles. In this review, we will discuss the signaling and metabolite exchanges between the ER and other organelles during abiotic stress responses in plants as well as the ER-organelle membrane contact sites and their associated tethering complexes.

## Introduction

Plants growing under natural habitats have to deal with various environmental stresses during their growth and development. Abiotic stresses such as extreme cold and hot temperatures, drought, salinity, and nutrient deficiency can greatly affect plant growth and crop productivity. Plants have evolved various sophisticated strategies to respond to different environmental stimuli at different levels from alternations in gene expression to changes in morphology (Nakashima et al., 2009; Su et al., 2013). The sensing and transduction of the environmental signals in stressed plants were intensively studied in the past several decades, revealing potential strategies to improve plant stress tolerance and agricultural productivity. It is generally believed that plant cells sense external environmental stimuli by various sensors, which are localized on the plasma membrane (PM), in the cytosol, or inside organelles. These environmental sensors activate intracellular signaling cascades that involve Ca^2+^, lipids, reactive oxygen species (ROS), and phytohormones (Osakabe et al., 2013; Zhu, 2016), ultimately inducing changes in gene expression, protein production, and metabolic pathways to enhance plant stress tolerance. Therefore, coordinated signaling between various intracellular compartments with distinct biochemical processes plays an important role in maintaining cellular homeostasis for the plant stress tolerance.

**Figure 1:**
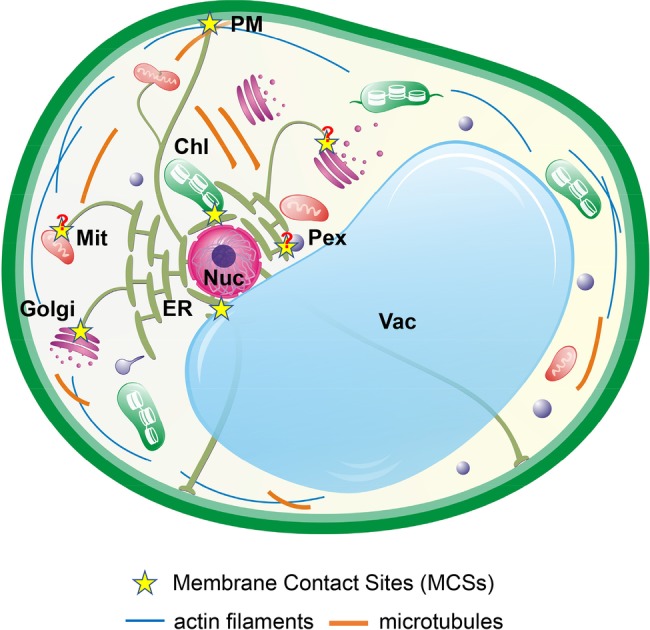
Interactions of the ER network with other organelles in plant cells. The dynamic ER network physically interacts with other subcellular compartments, such as the Golgi (*cis-* and *trans-*), mitochondria (Mit), chloroplasts (Chl), peroxisomes (PEX), vacuole (Vac), nucleus (Nuc), and the plasma membrane (PM) through MCSs. The pointed extensions of a peroxisome and a chloroplast represent peroxule and stromules, respectively. Question marks indicate MCSs that have not yet characterized. MCS-enriched proteins are directly involved in physical tethering; mediate organelle biogenesis; and regulate exchanges of lipids, Ca^2+^, ROS, and other important metabolites and signaling molecules.

The endoplasmic reticulum (ER) is a central network of interconnected tubules and flattened cisternae that extend throughout the entire cytoplasm of the eukaryotic cells (Figure 1). The ER network occupies a large volume of the cytoplasm, with its membrane accounting for ~50% of total cellular membranes, and functions in protein processing and folding, lipid biosynthesis, and Ca^2+^ storage (Stefano and Brandizzi, 2018). In eukaryote cells, about one-third of newly synthesized proteins enter the ER where they are glycosylated, folded, and/or assembled into protein complexes. The ER houses several stringent quality control mechanisms that export only correctly folded and properly assembled proteins to continue their secretory journeys (Hetz et al., 2015). However, protein folding in the ER is an error-prone process that could easily be disturbed by various abiotic and biotic stresses, leading to accumulation of mis/unfolded proteins in the ER and causing ER stress (Angelos et al., 2017). Currently, the unfolded protein response (UPR) is widely considered as a significant intracellular signaling pathway that links the ER proteostasis with gene regulation in the nucleus to alleviate the ER stress. Given its characteristic dynamic architecture and its essential roles in producing proteins and lipids for other organelles and maintaining Ca^2+^ homeostasis, the ER makes numerous physical contacts with other organelles and endomembrane compartments (Figure 1; Stefano and Brandizzi, 2018; Wu et al., 2018). Recent studies have identified many so-called ER-membrane contact sites (MCSs) that facilitate exchanges of important metabolites and signaling molecules between the ER and various organelles (Prinz, 2014; Wang and Dehesh, 2018; Wu et al., 2018). In this review, we will discuss recent results on the inter-organellar communications between the ER and other organelles during plant abiotic stress responses as well as the ER-organelle physical contacts and their associated tethering complexes.

## The Endoplasmic Reticulum-Nucleus Interaction *via* Unfolded Protein Response

In addition to the physical ER-nuclear envelop connection (Figure 1), the ER-nucleus interaction is mediated by a highly conserved signaling mechanism known as UPR, which is activated by accumulation of misfolded proteins in the ER. Because protein folding is an error-prone process that can easily be disturbed by various environmental stresses, UPR is closely connected to the plant stress tolerance (Liu and Howell, 2016). In plants, the UPR pathway is principally mediated by two major branches that are conserved in mammalian cells (Howell, 2013). One arm is mediated by two homologous ER membrane-anchored bZIP-family transcription factors, bZIP17 and bZIP28 that are activated by regulated intradomain proteolysis (Liu et al., 2007a). bZIP17 was originally identified as a transcription factor activated by salt stress (Liu et al., 2007b), while bZIP28 was discovered to be activated by heat stress (Gao et al., 2008). Both bZIP17 and bZIP28 are type II transmembrane proteins with a single transmembrane domain (TMD) and a DNA-binding/transcriptional activation bZIP domain facing the cytosol, and a C-terminal domain inside the ER lumen (Sun et al., 2013). In response to ER stress, bZIP17 and bZIP28 dissociate from the major ER luminal chaperone, binding immunoglobulin proteins (BiPs), and traffic from the ER to the Golgi where the two bZIP proteins are proteolytically processed by the Golgi-resident site-1 and site-2 proteases (S1P and S2P), thus releasing their N-terminal cytosolic domains that move into the nucleus (Andersson et al., 2007; Liu et al., 2007b; Gao et al., 2008; Srivastava et al., 2012; Iwata et al., 2017). The nuclear-localized bZIP17/28 proteins bind to their target promoters to increase expression of genes encoding ER chaperones, folding catalysts, and components of the ER-associated degradation (ERAD) machinery, which work together to restore the ER homeostasis (Liu and Howell, 2010). Interestingly, high light intensity increases ER stress sensitivity of plants *via* a competitive inhibitory interaction of bZIP28 with LONG HYPOCOTYL5 (HY5), a bZIP protein that positively regulates light signaling but suppresses the UPR pathway (Nawkar et al., 2017). The other arm of the plant UPR pathway involves the unconventional splicing of the mRNA of another bZIP transcription factor, bZIP60, which is catalyzed by the ER membrane-anchored inositol-requiring enzyme 1 (IRE1) (Deng et al., 2011; Nagashima et al., 2011). IRE1 is the most conserved ER stress sensor among yeast, plants, and animals and is a dual-functional protein with both protein serine/threonine kinase and endoribonuclease (RNase) activities. *Arabidopsis* has two IRE1 homologs, IRE1a and IRE1b (Koizumi et al., 2001). Under the ER stress, IRE1a and IRE1b can form homodimers or heterodimers to trigger their RNase activities, which splice the *bZIP60* mRNA (Howell, 2013). The frame-shift splicing of the *bZIP60* mRNA causes production of the active form of bZIP60 (bZIP60s, s for spliced) that lacks a transmembrane domain and can thus move into the nucleus to bind promoters of its target genes (Deng et al., 2011; Nagashima et al., 2011; Iwata and Koizumi, 2012). In addition to its *bZIP60* mRNA splicing role, the *Arabidopsis* IRE1s also participate in selective degradation of certain mRNAs of secretory pathway proteins and inhibitory proteins of the ER stress-induced autophagy, a process known as regulated IRE1-dependent decay of mRNAs (RIDD) (Mishiba et al., 2013; Bao et al., 2018). In plants, the ER stress responses are closely related to abiotic stress tolerance. *Arabidopsis* mutants defective in bZIP17, bZIP28, and/or bZIP60 show increased sensitivity to various environmental stresses whereas overexpression of the active forms of the three bZIP proteins enhances the plant stress tolerance (Fujita et al., 2007; Liu et al., 2007b; Kataoka et al., 2017; Ruberti et al., 2018). A recent study also implicated bZIP17 and a component of the *Arabidopsis* ERAD machinery in salt acclimation memory that enables plants to tolerate severe salt stress (Tian et al., 2018).

## The Endoplasmic Reticulum-Golgi Relationship

The ER and the Golgi apparatus are the first two membrane compartments in the protein secretory pathway. Unlike the mammalian cells in which the ER and the Golgi apparatus are separated by the ER-Golgi intermediate compartment (ERGIC, also known as the vesicular-tubular cluster or VTC), the ER and the Golgi complex are often physically attached in plant cells at ER exit sites (ERES) (Figure 1; Sparkes et al., 2009), although recent studies suggested the presence of an ERGIC-like compartment termed as GECCO for Golgi entry core compartment in plant cells (Ito et al., 2012, 2018). The ER-Golgi interaction involves the coat protein complex II (COPII)-mediated cargo export from the ER and the COPI-mediated retrieval of ER-resident proteins from the Golgi. Due to the existence of high stringent quality control mechanisms, only the correctly folded and properly assembled proteins can be exported from the ER into the Golgi, whereas those incompletely-/misfolded and improperly assembled proteins are retained in the ER for chaperone-assisted refolding or removal by ERAD that involves cytosolic proteasomes (Brandizzi and Barlowe, 2013; Liu and Li, 2014). In the Golgi complex that includes the *trans*-Golgi network (TGN), the ER-derived protein cargos undergo N-glycan maturation and are sorted by vesicle-dependent/independent trafficking pathways to specific destinations to carry out their cellular functions. Live cell imaging revealed that the plant Golgi apparatus is a highly dynamic organelle with dispersed stacks of cisternae that are often physically attached to the ER tubules (Figure 1; Sparkes et al., 2009). Additionally, the shape and architecture of the Golgi complex are flexible enough to adapt to the functional status of different plant cells (Dupree and Sherrier, 1998). These functional and physical connections between the ER and the Golgi complex not only ensure normal cellular activities but are also essential for the survival of plant cells during stress conditions.

Recent studies have shown that several *Arabidopsis* mutants deficient in the ER-Golgi/Golgi-ER vesicle trafficking exhibit the ER stress and are hypersensitive to abscisic acid (ABA) and salt stress (Zhao et al., 2013, 2018; Pastor-Cantizano et al., 2018), suggesting that the bidirectional vesicle transport between the ER and Golgi is crucial for maintaining cellular homeostasis and adaptation to environment stresses. In addition to vesicular trafficking, accumulating evidence indicates the existence of non-vesicular transport connecting the ER and Golgi. Three-dimensional electron microscopy and Forster resonance energy transfer-based fluorescence lifetime imaging microscopy revealed the physical contacts between the ER subdomains and *trans*-Golgi/TGN in mammalian cells (Ladinsky et al., 1999; Venditti et al., 2019b). No ER-*trans*-Golgi/TGN (referred hereinafter as ER-TG) contact has been observed so far in plant cells, but laser trap was used to reveal the ER-*cis*-Golgi interaction in plant cells, which occurs at ERES where the mobile Golgi stacks are associated with COPII components (Figure 1; Dasilva et al., 2004; Hawes et al., 2008; Sparkes et al., 2009). AtCASP, a homolog of a yeast/mammalian transmembrane Golgi protein known as CCAAT-displacement protein alternatively spliced product (CASP) was recently identified as a component of a novel tethering complex that connects ERES with the *cis*-Golgi to form the so-called “mobile secretory unit” (Osterrieder et al., 2017). The *cis*-Golgi-localized AtCASP could interact with ERES-enriched proteins to mediate the ER-*cis*-Golgi tethering that likely increases the efficiency of COPII vesicle-mediated cargo transport *via* the so-called “hug-and-kiss” mechanism (Kurokawa et al., 2014). Identification of potential AtCASP-binding proteins that are enriched at ERES could discover additional components of the ER-*cis*-Golgi tethering complex that might help to resolve the controversy on the mechanism of the ER-Golgi transport (Robinson et al., 2015) and explain the “sticky” nature of the plant *cis*-Golgi cisterna (Sparkes et al., 2009).

Mammalian cells lack the ER-*cis*-Golgi physical contact but contain multiple ER-TG contact sites that are implicated in the non-vesicle-mediated lipid exchange (Figure 1; Mesmin et al., 2019; Venditti et al., 2019b). Several lipid transfer proteins (LTPs) localized at the ER-TG interface were identified, such as CERT (ceramide-transfer protein), FAPP2 (four-phosphate adaptor protein 2), and OSBP (oxysterol-binding protein), which mediate the vesicle-independent ER-TG transport of ceramide, glucosylceramide, and cholesterol (coupled with counter-transport of phosphatidylinositol-4-phosphate), respectively (Mesmin et al., 2019; Venditti et al., 2019a). All three LTPs share similar protein domains important for the ER-TG bridging, including a TGN-binding N-terminal pleckstrin homology (PH) domain, a central FFAT (diphenylalanine in an acidic tract) motif exhibiting specific binding to the ER-localized vesicle-associated membrane protein-associated proteins (VAPs), and the C-terminal oxysteroid-binding domain. Almost nothing is known about the ER-TG contact in plant cells, but the *Arabidopsis* genome encodes multiple homologs of CERT/FAPP2/OSBP (Umate, 2011) that lack the FFAT motif and a total of 12 VAP homologs known as plant VAP homologs (PVAs) (Sutter et al., 2006). One of the *Arabidopsis* OSBP-related proteins (ORPs), ORP3a, is localized to the ER *via* its interaction with an ER-localized PVA, PVA12 through a WFDE (tryptophan-phenylalanine-aspartate–glutamate) motif located on the surface of ORP3a (Saravanan et al., 2009). It remains to be investigated whether or not plant cells have the ER-TG physical contacts, and if so, whether some of the *Arabidopsis* homologs of CERT/FAPP2/OSBP interact with ER-localized PVAs to mediate the ER-TG tethering and the ER-TG lipid/sterol exchanges.

## The Endoplasmic Reticulum-Mitochondria Connection

Mitochondrion is an intracellular double-membrane organelle found in all eukaryotic cells. It not only provides cellular energy and metabolic intermediates but also participates in many other cellular processes, such as ROS signaling, Ca^2+^ buffering, cell differentiation, and apoptosis (Labbé et al., 2014). Under changing environmental conditions, plants have to adjust their metabolism to balance their energy production and consumption through mitochondria. Recently, a growing body of evidence suggests that mitochondria and the ER cooperate in several biosynthetic pathways and exchange signaling molecules during stress conditions (Mueller and Reski, 2015; Wang and Dehesh, 2018). It is well known that environmental stresses, such as heat, drought, salinity, and high light intensity, increase production and accumulation of ROS in mitochondria, which not only serves an important intracellular signal (at low concentrations) to regulate various cellular pathways but also causes oxidative damage (at high concentrations) to the cellular components (Suzuki et al., 2012; Das and Roychoudhury, 2014). ROS can also be generated in the ER lumen, which has a higher redox potential (~100 mV) than that of other cellular compartments (Birk et al., 2013). The oxidative protein folding process in the ER is mediated by protein disulfide isomerases (PDIs) and a flavin adenine dinucleotide-binding protein, ER oxidoreductase 1 (Ero1), which produces H_2_O_2_ as a result of electron flow from target proteins *via* the PDI-Ero1 couple to O_2_ (Tu and Weissman, 2002; Santos et al., 2009; Higa and Chevet, 2012). Due to the H_2_O_2_ permeability of the ER membrane (Ramming et al., 2014), the ER-induced oxidative stress can influence the production of mitochondrial ROS likely mediated by the ER-mitochondria physical contacts (Bhandary et al., 2012; Murphy, 2013; Zeeshan et al., 2016). On the other hand, the mitochondrial ROS can induce expression of the ER UPR target genes (Ozgur et al., 2015).

The ER-mitochondria contact is also essential to build the membrane system of mitochondria that import most lipids from other organelles (Li-Beisson et al., 2017). The ER-mitochondria tethering allows lipid exchanges between two apposed membranes and/or permits access of the membrane-localized enzymes to lipid substrates on the tethered membrane (Michaud et al., 2017). In yeast, the ER-mitochondria encounter structure (ERMES) is the most well-defined ER-mitochondria tethering complex that facilitates the ER-mitochondria phospholipid exchanges (Figure 1; Michel and Kornmann, 2012; Lang et al., 2015). The yeast ER-mitochondria tethering also involves another complex known as the ER membrane complex (EMC)-translocase of outer membrane 5 kDa subunit (TOM5) complex (Lahiri et al., 2014). In mammalian cells, the ER-mitochondria interface, known as mitochondria-associated ER membrane (MAM), has more complicated protein complexes involved in physical tethering, Ca^2+^ regulation, lipid exchanges, mitochondrial fission, autophagy, and apoptosis (Lee and Min, 2018). In plants, despite visual evidence for the ER-mitochondria physical interaction that likely plays a role in mitochondrial fission and the ER-mitochondria coordinated biosynthesis and exchanges of phospholipids (Figure 1; Mueller and Reski, 2015; Michaud et al., 2017), no homologs of the yeast ERMES were found in plants that also lack homologs of a majority of known mammalian MAM proteins (Duncan et al., 2013; Michaud et al., 2017). The *Arabidopsis* genome does encode homologs of three of the six components (EMC1, 2, 3, 5, 6, and TOM5) of the EMC-TOM5 complex (Michaud et al., 2016) and homologs of mitofusin1 (MFN1), a mitochondrial fusion GTPase that interacts with its ER-localized homolog MFN2 to mediate the ER-mitochondria tethering (Detmer and Chan, 2007; de Brito and Scorrano, 2008). However, the two *Arabidopsis* MFN1/2 homologs, DRP3A/3B and FZL, are not involved in mitochondrial fusion (Arimura, 2018), and there is no report on the involvement of the three homologs of the yeast EMC-TOM5 complex in the ER-mitochondria tethering in plant cells. A recent study identified a *Physcomitrella patens* protein, MELL1 (mitochondria-ER-localized LEA-related LysM domain protein 1) that regulates the numbers of the ER-mitochondria contact sites and could thus be a component of the plant ER-mitochondria tethering complex (Mueller and Reski, 2015). It will be interesting to determine whether MELL1 is conserved in higher plants and if so, whether the MELL1 homologs are a component of the yet to be identified ERMES/MAM in higher plants and required for the phospholipid biosynthesis/exchange of the ER and mitochondria. The lipid exchanges between the ER and mitochondria also involve lipid trafficking between the inner membrane (IM) and outer membrane (OM) of the mitochondria. A recent study implicated a mitochondrial transmembrane lipoprotein (MTL) complex containing the TOM complex and IM-localized AtMIC60, an *Arabidopsis* homolog of the yeast MIC60 that is a component of the well-studied mitochondria contact site and cristae organizing system (MICOS) (Pfanner et al., 2014), in the IM-OM lipid trafficking (Michaud et al., 2016). It is thus possible that the TOM complex, through its interaction with IM-localized AtMIC60 capable of extracting membrane lipid and the ER-localized homologs of the yeast EMC-TOM5 complex, functions as a crucial component of a plant ER-mitochondria tethering complex to mediate lipid exchanges or coordinate lipid biosynthesis.

The ER-mitochondria physical contact is also essential for the Ca^2+^ cross talk between the two organelles, which is often influenced by ROS. In plants, a variety of environmental stimuli trigger Ca^2+^ transients, such as the influx of Ca^2+^ into the mitochondrial matrix, to regulate gene expression and metabolism (Carraretto et al., 2016). However, the ER is generally considered the main intracellular Ca^2+^ store. The Ca^2+^ channels located at the ER-mitochondria contact sites, such as the mitochondrial outer membrane-localized VDAC (voltage-dependent anion-selective channel) and the ER membrane-anchored inositol triphosphate-dependent calcium channel IP_3_R, are believed to mediate the transport of Ca^2+^ between the ER and mitochondria in response to ER stress in mammalian cells (Lee and Min, 2018). The mammalian ER-localized Ca^2+^-release channel ryanodine receptor is activated by Ero1-generated H_2_O_2_ (Anelli et al., 2012). It remains to be determined if the ER ROS also regulates the ER Ca^2+^ release in plant cells that lack the homologs of the mammalian ER Ca^2+^ efflux channels IP_3_R and ryanodine receptor (Stael et al., 2012).

Two recent studies revealed another interesting mechanism by which the ER interacts with the mitochondria in plant cells. The mitochondrial retrograde regulation (MRR), which transmits the stress-induced mitochondrial signal into the nucleus to increase production of certain mitochondrial proteins for sustaining or restoring the mitochondrial functions during stressful conditions (Dojcinovic et al., 2005), was shown to involve two ER-anchored NAC transcription factors, ANAC013 and ANAC017 (De Clercq et al., 2013; Ng et al., 2013). *ANAC013* knockdown lines and an *ANAC017* knockout mutant were hypersensitive to stress than their wild-type controls. It was hypothesized that the mitochondrial stress somehow activates yet unknown proteases that proteolytically activate the two ER-anchored ANAC proteins that can subsequently translocate into the nucleus (Wang et al., 2018c). It will be interesting to test if the proteolytic activation of the two NAC-type transcription factors occurs at ERMES/MAM in plant cells. Proteomic experiments with stressed *Arabidopsis* plants expressing non-cleavable variants of ANAC013/017 might lead to identification of potential components of the *Arabidopsis* ERMES/MAM. It is also interesting to note that the two ANACs were recently implicated in coordinating mitochondrial and chloroplast functions *via* their physical interactions with a nuclear protein Radical-induced Cell Death1 (RCD1) that was known to be regulated by ROS (Shapiguzov et al., 2019).

## The Endoplasmic Reticulum-Plasma Membrane Contact

The plasma membrane (PM), a lipid bilayer embedded with proteins, is an essential cellular component for the plant stress tolerance. It not only serves as a physical barrier to shield cellular contents from the extracellular environment and controls the flux of solutes and macromolecules but also contains a wide range of sensors and receptors that perceive and transmit all kinds of environmental signals. As discussed above, the ER not only produces, folds, and assembles the PM-localized channels/transporters and receptors/sensors but also delivers lipids to the PM and other intracellular compartments *via* vesicle-dependent and/or independent mechanisms.

The ER-PM contact sites (EPCSs) are evolutionarily conserved microdomains that are important for the ER-PM communications, such as lipid homeostasis, and Ca^2+^ influx (Figure 1; Saheki and De Camilli, 2017). The composition of EPCSs and their molecular functions have been well established in the yeast and mammalian cells in the last decade (Stefan, 2018). The yeast EPCSs consists of six proteins: three tricalbins, Increased sodium tolerance protein 2 (Ist2), and the ER-resident protein Scs2/22 (Suppressor of choline sensitivity 2/22) (Manford et al., 2012). The mammalian EPCSs contains three tricalbin homologs known as E-Syts for extended synaptotagmin (Giordano et al., 2013) and two Scs2/22 homologs, VAP-A and VAP-B, but lacks an Ist2 homolog (Selitrennik and Lev, 2016). In plants, the EPCS complex is the best known protein tether of the plant ER MCSs and consists of VAP27, VAP-Related Suppressor of TMM (VST), an actin-binding protein NETWORKED 3C (NET3C), actin filaments, and microtubule networks (Figure 1; Wang et al., 2014, 2016, 2017, 2018a; Ho et al., 2016). In particular, a phospholipid-binding protein Synaptotagmin1 (SYT1), which is the plant homolog of tricalbin/E-Syts, was found in the plant EPCS complex (Perez-Sancho et al., 2015) and subsequently used as a marker for the plant EPCS for microscopic studies (McFarlane et al., 2017; Lee et al., 2019). SYT1 has been previously described as an essential component for maintaining the PM integrity, especially under conditions of high risks of membrane disruption such as osmotic shock, freezing, and salt stresses (Schapire et al., 2008). Other studies have shown that SYT1 is required for tethering the ER to the PM and plays an essential role in regulating the ER remodeling and the stability of EPCSs (Siao et al., 2016). A recent study revealed that the ER-anchored SYT1 directly binds the PM-localized phosphatidylinositol 4,5-bisphosphate [PI (4,5)P2] to establish EPCSs (Lee et al., 2019), thus revealing a physiological function of the stressed-induced PM accumulation PI(4,5)P2 (Heilmann, 2008). It is likely that the protein-lipid tether could be disrupted or strengthened by additional SYT1/PI(4,5)P2-binding proteins.

EPCSs are now widely accepted as important sites for the non-vesicular lipid transport, which appears to be the major transport route of certain lipid species (Lev, 2012). Plants exposed to abiotic stresses have to adapt their membrane lipid composition and fluidity to changing environmental conditions by adjusting the relative amounts of various lipids, such as phospholipids and galactolipids (Hou et al., 2016). It is well known that lipids synthesized in the ER need to be delivered to other membranes for assembly of biological membranes or for lipid-mediated signaling cascades. It is proposed that the lipid transfer proteins (LTPs) are localized at the EPCSs and function as dynamic tethers between the two membranes with their lipid transfer module regulating lipid exchange (Dickson et al., 2016; Quon et al., 2018). Mammalian VAPs are known to interact with proteins involved in lipid transfer (Gatta et al., 2015) while SYT1 contains a synaptotagmin-like mitochondria-lipid-binding protein (SMP) domain that is implicated in lipid transfer in mammals (Schauder et al., 2014). It is likely that the plant EPCSs are also involved in the ER-PM lipid transfer and thus play important role in plant stress tolerance by modulating the composition and fluidity of the PM. The EPCS is also important for the intracellular Ca^2+^ homeostasis in mammalian cells. The ER-PM contacts are critically implicated in generating the cytosolic Ca^2+^ signals, which is likely mediated by Ca^2+^ release from the ER in response to the PM-perceived environmental stimuli, and in replenishing the depleted ER Ca^2+^ store (Chung et al., 2017). Given the importance of Ca^2+^ signaling in plant stress response (Ranty et al., 2016), it would be interesting to investigate the role of EPCSs in regulating the stress-triggered intracellular Ca^2+^ dynamics in plants.

In addition to the EPCS-mediated exchange of lipids and Ca^2+^, there are other mechanisms that connect the ER physiology to the PM function in plant stress response. A recent study implicated a PM-localized NAC transcription factor, ANAC062, in the ER-nucleus-mediated UPR pathway (Yang et al., 2014). It is quite possible that the ER stress could increase the EPCS formation, altering the local membrane lipid composition to enhance the proteolytic processing of the PM-anchored ANAC062 (Seo et al., 2010). The cleaved ANAC062 can then move into the nucleus to regulate UPR-related genes, thus helping to mitigate the ER stress. Other studies found that the increased cytosolic Ca^2+^ caused by the stress-triggered Ca^2+^ release from the ER could activate the PM-localized NADPH oxidase, which was known to be induced by UPR and is required to survive ER stress (Ozgur et al., 2015, 2018; Angelos and Brandizzi, 2018). It is quite tempting to speculate that the Ca^2+^-mediated activation of the PM-localized NADPH oxidase might require EPCSs. It is important to note that the plant NADPH oxidase is the most well-studied ROS enzymatic system and plays a key role in ROS signaling involved in plant growth, stress tolerance, and plant immunity (Marino et al., 2012).

One unique type of the plant ER-PM contact occurs at plasmodesmata (PD), which consist of the cylindrically apposed PM and the tightly compressed ER (desmotubule) with unique lipid/protein compositions (Grison et al., 2015; Leijon et al., 2018). The PD-PM and the desmotubule are connected by spoke-like elements (Ding et al., 1992; Nicolas et al., 2017) whose molecular identities remain to be defined, but recent studies suggested the PD association of AtSYT1 (Levy et al., 2015) and VAP27 (Wang et al., 2016). The space between the PD-PM and the desmotubule constitutes the actual channel (the cytoplasmic sleeve) that transports a wide range of molecular cargos across cell walls of neighboring cells (Tilsner et al., 2016). Given the key role of PD in generating cytosolic and membrane continuity that are essential for growth and development, stress tolerance, and plant defense, the permeability of PD (also known as size exclusion limit), governed by the size of the cytoplasmic sleeve and distribution of spokes that creates nanochannels, is constantly regulated by various of developmental and environmental signals (Sun et al., 2019). Although PD exhibits the essential features of MCS (Scorrano et al., 2019), it remains to be investigated if the ER-PM contacts in PD play any role in inter-organelle exchanges of lipids, Ca^2+^, and/or other signaling molecules.

## The Endoplasmic Reticulum-Chloroplast Junction

Chloroplasts conduct photosynthesis and produce energy for plant growth, development, and defense. In addition, chloroplasts are essential for synthesizing certain amino acids, lipids, and fatty acids. Like mitochondrion, chloroplast is also a semiautonomous organelle with its own genome and a majority of chloroplast proteins are encoded by the nuclear genome and imported from the cytosol. Accordingly, the plant cells execute anterograde and retrograde communications between the chloroplast and the nucleus to respond to changing environment (Watson et al., 2018). Under stress conditions, ROS such as singlet oxygen and superoxide were generated from electron transport chain in the chloroplasts, which cause oxidative damage to the photosynthetic organelle. Consequently, the chloroplasts use ROS and several metabolites, such as 3′-phosphoadenosine 5′-phosphate (PAP) (Chan et al., 2016) and methylerythritol cyclodiphosphate (MEcPP) (Xiao et al., 2012), to relay the stress signal into the nucleus to reprogram gene expression for damage mitigation and stress acclimation (Woodson and Chory, 2012). The chloroplast-nucleus signaling might also involve chloroplast-nucleus contact sites consisting of stromules, the stroma-filled tubular protrusions from the chloroplast outer membrane (Figure 1; Kohler and Hanson, 2000; Hanson and Hines, 2018), which facilitate translocations of chloroplast-sequestered transcription factors into the nucleus in response to various stresses (Caplan et al., 2008; Sun et al., 2011; Foyer et al., 2014). Stromules were also known to be associated with the ER, Golgi apparatus, PM, mitochondria, and peroxisomes (Kwok and Hanson, 2004; Schattat et al., 2011; Hanson and Hines, 2018); however, the physiological significance of these associations remains to be investigated in the coming years.

The ER and chloroplasts are the two major sites of lipid biosynthesis (van Meer et al., 2008; Hurlock et al., 2014) and the ER-chloroplast interaction is essential for lipid homeostasis in plant cells under normal growth condition and in response to various environmental stresses (Negi et al., 2018; Lavell and Benning, 2019). The ER-chloroplast-mediated lipid biosynthesis involving *de novo* synthesis of fatty acids (FAs) in chloroplasts, the chloroplast-ER transport of FAs, the ER-catalyzed assembly and modification of glycerolipids that move back to chloroplasts for producing galactolipids (Benning and Ohta, 2005), the major chloroplast lipids (Dormann and Benning, 2002). Studies in recent years strongly suggest that the chloroplast-ER physical contact sites, better known as plastid-associated membranes [PLAMs, (Andersson et al., 2007)], are directly involved in the lipid exchange (Tan et al., 2011; Block and Jouhet, 2015). At least two groups of proteins were detected at the ER-chloroplast membrane contact sites (Tan et al., 2011). The first group includes several members of the trigalactosyldiacylglycerol (TGD) protein family, which form a bacterial-type ABC transporter for transporting lipids from the ER to the thylakoid membrane (Xu et al., 2010; Wang et al., 2012; Fan et al., 2015). The second group includes lipid processing enzymes such as phosphatidylcholine (PC) synthase and CLIP1 lipase/acylhydrolase that directly act on lipids from the contacting ER-chloroplast membranes (Mehrshahi et al., 2013, 2014). In addition, a recent study indicated the presence of several lipid transfer proteins, including Azelaic Acid Induced 1 (AZI1), EArly *Arabidopsis* Aluminum Induced 1 (EARLI1), and Defective in Induced Resistance 1 (DIR1), at the ER-chloroplast contact site that facilitates the movement of a lipid-derived signal for systemic acquired resistance against pathogens (Cecchini et al., 2015).

Various abiotic stresses, such as high light exposure and wounding, can lead to accumulation of MEcPP in chloroplasts, which serves as a retrograde signaling metabolite that relays the chloroplast stress signal into the nucleus to alter gene expression (Xiao et al., 2012). Intriguingly, the chloroplast-synthesized MEcPP signal could activate the transcription of IRE1 and bZIP60, two key components of the ER stress-triggered UPR pathway *via* a Ca^2+^-dependent transcription factor calmodulin-binding transcription activator3 (Walley et al., 2015; Benn et al., 2016). In addition, a loss-of-function mutation in an *Arabidopsis* gene encoding the chloroplast stearoyl-acyl carrier protein desaturase, which introduces double bonds into FAs, constitutively activates the expression of a known ER-UPR marker gene *BIP3* (Iwata et al., 2018). A loss-of-function mutation in the *Arabidopsis SAL1* gene, which encodes a chloroplast/mitochondria-localized bifunctional enzyme with both 3′(2′),5′-bisphosphate nucleotidase (converting PAP to AMP) and inositol polyphosphate 1-phosphatase activities, attenuated ER stress response and exhibited hyposensitivity to ER stress inducers (Xi et al., 2016). Together, these findings provide additional support for the involvement of the photosynthetic organelle in regulating the ER homeostasis.

## The Endoplasmic Reticulum-Peroxisome Collaboration

Peroxisome is a semiautonomous single-membrane-bound organelle that participates in a wide range of biochemical processes, particularly the β-oxidation of fatty acids and metabolism of hydrogen peroxide (Smith and Aitchison, 2013). In plants, peroxisomes also perform other important functions such as the glycolate cycle and photorespiration, secondary metabolism, hormone (auxin and jasmonic acid) biosynthesis, metabolism of ROS and reactive nitrogen species (RNS) (Nyathi and Baker, 2006; Hu et al., 2012; Sandalio and Romero-Puertas, 2015). Notably, peroxisomes are highly dynamic organelles that alter their morphology, proliferation, and metabolic activities in response to environmental signals (Honsho et al., 2016; Kao et al., 2018). The membrane extensions of peroxisomes, termed as peroxules (Figure 1), are often observed when plants are exposed to exogenous H_2_O_2_ or high-intensity light (Sinclair et al., 2009; Barton et al., 2013; Jaipargas et al., 2016). Salt stress, heavy metals, and herbicide application were known to increase the metabolic activity and proliferation rate of peroxisomes (Palma et al., 1987; McCarthy et al., 2001; Mitsuya et al., 2010; McCarthy-Suárez et al., 2011; Fahy et al., 2017).

It has been well known that peroxisome dynamics such as elongation, fission, and degradation as well as metabolic changes require their constant collaborations and communications with other intracellular organelles (Hu et al., 2012; Del Rio and Lopez-Huertas, 2016; Kao et al., 2018). The ER-peroxisome connection has been known for many years as peroxisomes are formed by budding from specialized ER regions and/or by growth and fission of preexisting peroxisomes in yeast and mammalian cells (Hu et al., 2012; Kao et al., 2018). Although there is no clear evidence to support the ER budding model for the plant peroxisomes (Mullen and Trelease, 2006; Trelease and Lingard, 2006), the ER is at least involved in the plant peroxisome biogenesis by providing membranes, lipids, and certain peroxisome membrane proteins (PMPs) to preexisting or fission-created nascent peroxisomes (Hu et al., 2012).

The plant peroxisomes were shown to be closely associated with the ER by early microscopic observation (Huang et al., 1983) and could be physically attached to the ER as suggested by live cell imaging of dynamic behaviors of peroxisomes (and peroxules) and the ER in *Arabidopsis* (Mathur, 2009; Sinclair et al., 2009; Barton et al., 2013). However, it remains unknown whether the observed ER-peroxisome contiguity in *Arabidopsis* is mediated by the peroxisome-ER physical tether that was first described in yeast. The yeast peroxisome-ER tethering complex consists of a peroxisome biogenic protein, peroxin 3 (PEX3), localized on the ER and peroxisome, and the peroxisome inheritance factor Inp1 that serves as a bridge to link the ER and peroxisome-localized PEX3 (Knoblach and Rachubinski, 2013). The PEX3-Inp1-PEX3 trimeric complex plays a key role in partitioning peroxisomes in dividing yeast cells and controlling the peroxisome population (Knoblach et al., 2013). The mammalian peroxisome-ER tether consists of the ER-localized VAPs and the PMPs with acyl-CoA binding domains (ACBDs) and is thought to regulate peroxisome proliferation and to facilitate the ER-peroxisome lipid exchange (Hua et al., 2017; Costello et al., 2017a,b). Despite microscopic observations of the ER-peroxule association (Sinclair et al., 2009; Barton et al., 2013), a plant peroxisome-ER tethering complex remains to be discovered. The identification of a peroxisome-ER tether is expected to shed light on the functional collaboration between the two dynamic organelles, especially the mechanisms of peroxisome biogenesis/maintenance and their dynamic responses to various environmental stresses.

It was recently suggested that peroxisomes, ER, and mitochondria could form a “redox triangle” that uses tethering complexes to assemble a hypothetical “redoxosome” that transmits intercompartmental redox signals to regulate ROS metabolism in response to cellular signals and environmental cues (Yoboue et al., 2018). A plant “redoxosome” should include protein tethering complexes of chloroplasts with the ER, mitochondria, and peroxisome. The chloroplast works together with mitochondria and peroxisomes in photorespiration involving inter-organellar metabolite exchanges while the chloroplast tubular extensions, stromules, are thought to interact with the ER, mitochondria, and peroxisomes (Mathur et al., 2012; Hanson and Hines, 2018). Fluorescent microscopic studies and proteomic experiments with a plant genetic model system such as *Arabidopsis* could make a significant contribution to our understanding of such a “redoxosome” in plants. Dynamic physical associations of multiple organelles aided by organelle extensions and tethering complexes might be a common cellular mechanism that facilitates exchanges of ROS/RNS, Ca^2+^, lipids, and other metabolites/signaling molecules to mount coordinated cellular responses to changing environment.

## The Endoplasmic Reticulum-Vacuole Association

Vacuoles are single-membrane-bound organelles that are filled with a wide range of inorganic ions and organic molecules (Figure 1). In plants, at least two types of vacuoles have been identified, including protein storage vacuoles (PSVs) and lytic vacuoles (LVs) (Paris et al., 1996; Zhang et al., 2014). PSVs usually serve as a warehouse for seed storage proteins that are synthesized in the ER during seed maturation, while LVs occur in the vegetative tissues and contain acidic contents and degradative enzymes with lysosome-like properties (Shimada et al., 2018). It has been shown that the vacuoles play crucial roles in storage of nutrients and metabolites, detoxification, pH homeostasis, and stress tolerance (Muntz, 2007; Viotti, 2014). Maintaining proper turgor pressure in vacuoles is required for morphological alterations of cells during plant development, and the rapid vacuolar uptake or unloading of various ions and metabolites allows plants to efficiently cope with environmental stresses. For instance, AtNHX1 is an *Arabidopsis* tonoplast-localized Na^+^/H^+^ antiporter that moves excessive Na^+^ from the cytosol into the vacuole, lowering the water potential of the vacuole and driving water flow into the cells to maintain plants’ growth under high salinity condition (Apse et al., 1999). It is well known that stomatal opening or closure is associated with vacuole morphology changes in guard cells, highlighting the important roles of vacuole in plant response to abiotic stresses, such as high temperature and drought (Gao et al., 2005; Tanaka et al., 2007; Bak et al., 2013).

Many vacuolar proteins and metabolites are synthesized and processed in the ER and transported to the vacuoles. One well-established pathway for vacuolar transport is the COPII-mediated vesicle trafficking from the ER to the Golgi and the post-Golgi transport that involves the plant TGN and the pre-vacuolar compartment (PVC, also known as MVB for multi-vesicular body) (Xiang et al., 2013; Brillada and Rojas-Pierce, 2017). Recent studies indicated the presence of a direct Golgi-independent ER-vacuole trafficking route involving the machinery of autophagy (Viotti et al., 2013; Michaeli et al., 2014), which degrades and recycles damaged/misfolded/aggregated proteins and defective/excessive intracellular organelles (Wang et al., 2018b). More importantly, autophagy is an integral part of the ER stress-triggered UPR. Under the ER stress, ER components bud from the ER and form autophagosome with the aid of appropriate cargo receptors, and the autophagosome subsequently fuses with the lytic vacuole to release the ER cargos for degradation *via* the classical macroautophagy pathway (Liu et al., 2012; Michaeli et al., 2014; Yang et al., 2016). A special process of autophagy, ER-phagy (Schuck et al., 2014) or reticulophagy (Liu et al., 2012), is activated to degrade damaged ER fragments when UPR fails to mitigate the ER stress. Further studies revealed that the ER stress-induced reticulophagy in *Arabidopsis* requires one of the ER-localized UPR sensor IRE1b but not bZIP60 (Liu et al., 2012).

Given the presence of a direct ER-vacuole trafficking route for transporting metabolites, proteins, and membranes in plant cells, it is quite possible that plant cells have multiple ER-vacuole contact sites that serve important cellular functions, especially when responding to environmental stresses. In yeast, the ER-vacuole contact site (Figure 1) [known as nuclear ER-vacuole junctions or NVJs (Pan et al., 2000)] has been well studied and is implicated in the biogenesis and transport of lipid droplets in response to metabolic stress (Hariri et al., 2018). The yeast NVJ is established by interaction between one of the two ER membrane proteins, Nvj1 and Ltc1 (lipid transfer at contact site1), and an armadillo repeat protein Vac8 that requires palmitoylation for its localization to the vacuolar membrane (Pan et al., 2000; Murley et al., 2015). The yeast NVJ tether also contains Nvj2, one of the seven SPM domain-containing proteins that are localized at MCSs, including three at ERMES and the remaining three (tricalbins) at EPCSs (Toulmay and Prinz, 2012). Despite essential roles of the vacuoles in plant growth, stress tolerance, and plant defense (Shimada et al., 2018), little is known about the plant ER-vacuole contact sites and their associated tethering complexes. *Arabidopsis* lacks a homolog of Nvj1 or Ltc1 but contains >100 armadillo repeat proteins (Sharma et al., 2014) and five tricalbin homologs known as AtSYTA-E or AtSYT1–5 (Craxton, 2004). Live cell imaging of fluorescently tagged ER/tonoplast-localized proteins coupled with optical tweezers (Sparkes, 2018) could reveal potential ER-vacuole contact sites and their dynamic changes in response to environmental stresses. Given the widespread occurrence of SMP-containing proteins at multiple MCSs in yeast and mammalian cells (Toulmay and Prinz, 2012), identification of a plant ER-vacuole tethering complex might be facilitated by confocal microscopic examination of fluorescently tagged AtSYT1–5 followed by biochemical studies of an AtSYT localized at the ER-vacuole contact sites.

## Conclusion

Accumulating evidence supports important roles of the ER-organelle interactions in plant stress tolerance, which involves exchanges of metabolites and signaling molecules at specialized MCSs with unique tethering complexes. Further studies that combine live cell imaging, proteomics, and plant genetics are needed to fully understand the composition and dynamic regulation of these MCSs in response to environmental changes and their additional physiological functions.

## Author Contributions

LL and JL discussed the writing plan, LL drafted the manuscript, and JL edited the manuscript.

### Conflict of Interest Statement

The authors declare that the research was conducted in the absence of any commercial or financial relationships that could be construed as a potential conflict of interest.
